# Singularity in the matrix of the coupled Gross-Pitaevskii equations and the related state-transitions in three-species condensates

**DOI:** 10.1038/s41598-017-06843-3

**Published:** 2017-07-26

**Authors:** Y. M. Liu, Y. Z. He, C. G. Bao

**Affiliations:** 1 Department of Physics, Shaoguan University, Shaoguan, 512005 P. R. China; 2 School of Physics, Sun Yat-Sen University, Guangzhou, P. R. China; 3 State Key Laboratory of Theoretical Physics, Institute of Theoretical Physics, Chinese Academy of Sciences, Beijing, 100190 China

## Abstract

An approach is proposed to solve the coupled Gross-Pitaevskii equations (CGP) of the 3-species BEC in an analytical way under the Thomas-Fermi approximation (TFA). It was found that, when the strength of a kind of interaction increases and crosses over a critical value, a specific type of state-transition will occur and will cause a jump in the total energy. Due to the jump, the energy of the lowest symmetric state becomes considerably higher. This leaves a particular opportunity for the lowest asymmetric state to replace the symmetric states as the ground state. It was further found that the critical values are related to the singularity of either the matrix or a sub-matrix of the CGP. These critical values are not arising from the TFA but inherent in the CGP, and they can be analytically expressed. Furthermore, a model (in which two kinds of atoms separated from each other asymmetrically) has been proposed for the evaluation of the energy of the lowest asymmetric state. With this model the emergence of the asymmetric ground state is numerically confirmed under the TFA. The theoretical formalism of this paper is quite general and can be generalized for BEC with more than three species.

## Introduction

Accompanying the progress in techniques, the research into the 2-species Bose-Einstein condensates (2-BEC) is gaining an increasing attention in recent years in both the experimental aspect^1–7^ and theoretical aspect^8–26^. Many distinguished features have been found, say, the existence of various phases, the critical value in the inter-species interaction and the related instability, the emergence of asymmetric ground state (g.s.)^9, 11, 12^, the appearance of vortex, and so on. The study for BEC with three species (3-BEC) has also started^27–30^. It is very interesting to see how the phenomena found in 2-BEC would recover in 3-BEC and whether new phenomena would emerge. Note that, for multiband superconductivity, the interband couplings among a set of different band condensates are important to the critical behavior of the system. Critical temperatures are thereby substantially affected (not determined alone by the Cooper-pair amplitude of a single band)^31–33^. Thus, it is reasonable to expect that the critical phenomena found in 2-BEC (say, a state-transition has been found to take place when the strength of the inter-species interaction arrives at a critical value^34^) might also be affected and new critical phenomena might emerge. Since the BEC with more than two species are experimentally achievable, it is meaningful to perform theoretical research at this stage.

This paper is dedicated to a primary theoretical study on the 3-BEC based on the coupled Gross-Pitaevskii equations (CGP). Although exact numerical solutions of the CGP are very valuable, it is not easy to extract the underlying physics simply via numerical results. In order to gain more insight into the physics, it is helpful to obtain approximate analytical solutions. Therefore, the Thomas-Fermi approximation (TFA) has been adopted. Under the TFA, we provide an approach for obtaining analytical solutions. Thereby the wave functions and the total energies can be obtained in an analytical form, and these quantities can relate directly to the parameters involved. This facilitates greatly related physical analysis. We found that the singularity of the (sub)matrix-of-equations is crucial to the behavior of the BEC. Specific state-transitions will be induced when the parameters vary and cross over a singular point of the matrix. This will be studied in detail below.

Furthermore, based on the analytical formalism and the singularity of the equations, effort is made to divide the whole parameter-space into zones, each supports a specific spatial configuration. This provides a primary frame for plotting the phase-diagrams in the future. Besides, a model for calculating the total energies of asymmetric states has also been proposed. The possibility of the emergence of asymmetric g.s. has been primarily evaluated.

The theoretical formalism of this paper is quite general and can be generalized for the K-BEC with K larger than 3.

## Hamiltonian and the coupled Gross-pitaevskii equations

We assume that the 3-BEC contains *N*_*S*_ *S*-atoms with mass *m*_*S*_ and interacting via ${V}_{S}={c}_{S}{\sum }_{i < i^{\prime} }\,\delta ({{\bf{r}}}_{i}-{{\bf{r}}}_{i^{\prime} })$, (*S* = *A*, *B* and *C*). The particle numbers are assumed to be huge (say, ≥10000). The inter-species interactions are ${V}_{SS^{\prime} }={c}_{SS^{\prime} }{\sum }_{i=1}^{{N}_{S}}{\sum }_{j=1}^{{N}_{S^{\prime} }}\,\delta ({{\bf{r}}}_{i}-{{\bf{r}}}_{j})$ with the strength *c*_*ss*′_. These atoms are confined by the isotropic harmonic traps $\frac{1}{2}{m}_{S}{\omega }_{S}^{2}{r}^{2}$ We introduce a mass *m*_*o*_ and a frequency *ω*. Then, *ħω* and $\lambda \equiv \sqrt{\hslash /({m}_{o}\omega )}$ are used as units for energy and length. The spin-degrees of freedom are assumed to be frozen. The total Hamiltonian is

1 $$\begin{array}{rcl}H & = & {H}_{A}+{H}_{B}+{H}_{C}+{V}_{AB}+{V}_{BC}+{V}_{CA}\\ {H}_{A} & = & \sum _{i=1}^{{N}_{A}}\,(-\frac{{m}_{o}}{2{m}_{A}}{\nabla }_{i}^{2}+\frac{1}{2}{\gamma }_{A}{r}_{i}^{2})+{V}_{A}\end{array}$$

where *γ*_*A*_ = (*m*_*A*_/*m*_*o*_)(*ω*_*A*_/*ω*)^2^. *H*_*B*_ and *H*_*C*_ are similarly defined.

We assume that no spatial excitations are involved in the g.s. Thus, each kind of atoms are fully condensed into a state which is most advantageous for binding. Accordingly, the total many-body wave function of the g.s. can be written as

2 $${\rm{\Psi }}=\prod _{i=1}^{{N}_{A}}\,\frac{{u}_{1}({r}_{i})}{\sqrt{4\pi }{r}_{i}}\prod _{j=1}^{{N}_{B}}\,\frac{{u}_{2}({r}_{j})}{\sqrt{4\pi }{r}_{j}}\prod _{k=1}^{{N}_{C}}\,\frac{{u}_{3}({r}_{k})}{\sqrt{4\pi }{r}_{k}}$$

where *u*_1_, *u*_2_ and *u*_3_ are for the A-, B-, and C-atoms, respectively.

In the set of the CGP, the one for *u*_1_ is

3 $$\begin{array}{l}(-\frac{{m}_{o}}{2{m}_{A}}{\nabla }^{2}+\frac{1}{2}{\gamma }_{A}{r}^{2}+{N}_{A}{c}_{A}\frac{{u}_{1}^{2}}{4\pi {r}^{2}}+{N}_{B}{c}_{AB}\frac{{u}_{2}^{2}}{4\pi {r}^{2}}\\ \quad +\,{N}_{C}{c}_{CA}\frac{{u}_{3}^{2}}{4\pi {r}^{2}}-{\varepsilon }_{A}){u}_{1}=0\end{array}$$

where *ε*_*A*_ is the chemical potential. Via cyclic permutations of the three indexes (*A*, *B*, *C*) together with (*u*_1_, *u*_2_, *u*_3_). From eq. (3) we obtain the other two for *u*_2_ and *u*_3_. It is emphasized that the normalization $\int \,{u}_{l}^{2}dr=1$ (*l* = 1, 2 and 3) is required.

## Formal solutions under the Thomas-Fermi approximation

Since *N*_*A*_, *N*_*B*_ and *N*_*C*_ are considered to be large, TFA has been adopted. The applicability of this approximation has been evaluated via a numerical approach given in refs 22 and 35 and will be discussed later. Under the TFA, the CGP become

4 $$\begin{array}{rcl}(\frac{{r}^{2}}{2}+{\alpha }_{11}\frac{{u}_{1}^{2}}{{r}^{2}}+{\alpha }_{12}\frac{{u}_{2}^{2}}{{r}^{2}}+{\alpha }_{13}\frac{{u}_{3}^{2}}{{r}^{2}}-{\varepsilon }_{1}){u}_{1} & = & 0\\ (\frac{{r}^{2}}{2}+{\alpha }_{21}\frac{{u}_{1}^{2}}{{r}^{2}}+{\alpha }_{22}\frac{{u}_{2}^{2}}{{r}^{2}}+{\alpha }_{23}\frac{{u}_{3}^{2}}{{r}^{2}}-{\varepsilon }_{2}){u}_{2} & = & 0\\ (\frac{{r}^{2}}{2}+{\alpha }_{31}\frac{{u}_{1}^{2}}{{r}^{2}}+{\alpha }_{32}\frac{{u}_{2}^{2}}{{r}^{2}}+{\alpha }_{33}\frac{{u}_{3}^{2}}{{r}^{2}}-{\varepsilon }_{3}){u}_{3} & = & 0\end{array}$$

where *α*_11_ = *N*_*A*_ *c*_*A*_/(4*πγ*_*A*_), *α*_22_ = *N*_*B*_ *c*_*B*_/(4*πγ*_*B*_), *α*_33_ = *N*_*C*_ *c*_*C*_/(4*πγ*_*C*_), *α*_12_ = *N*_*B*_ *c*_*AB*_/(4*πγ*_*A*_), *α*_21_ = *N*_*A*_ *c*_*AB*_/(4*πγ*_*B*_), *α*_13_ = *N*_*C*_ *c*_*CA*_/(4*πγ*_*A*_), *α*_31_ = *N*_*A*_ *c*_*CA*_/(4*πγ*_*C*_), *α*_23_ = *N*_*C*_ *c*_*BC*_/(4*πγ*_*B*_), *α*_32_ = *N*_*B*_ *c*_*BC*_/(4*πγ*_*C*_), they are called the weighted strengths (W-strengths) and they are related as *α*_12_ *α*_23_ *α*_31_ = *α*_21_ *α*_32_ *α*_13_. *ε*_1_ = *ε*_*A*_/*γ*_*A*_, *ε*_2_ = *ε*_*B*_/*γ*_*B*_, *ε*_3_ = *ε*_*C*_/*γ*_*C*_, they are the weighted energies for a single particle. Recall that there are originally 15 parameters (*N*_*S*_, *m*_*S*_, *ω*_*S*_, *c*_*S*_, *c*_*ss*′_). Their combined effects are fully represented by the nine *α*_*ll*′_ (only eight of them are independent). Thus, based on the W-strengths, related analysis could be simpler. In this paper all the interactions are considered as repulsive. Accordingly, all the W-strengths are positive. Furthermore, it is safe to assume that all the three *u*_*l*_/*r* ≥ 0 (because they do not contain nodes).

The set of W-strengths forms a matrix ${\mathfrak{M}}$ (i.e., the matrix-of-equations) with matrix elements ${({\mathfrak{M}})}_{ll^{\prime} }={\alpha }_{ll^{\prime} }$. The determinant of ${\mathfrak{M}}$ is denoted by ${\mathfrak{D}}$. The set of equation (4) has four forms of formal solutions, each would hold in a specific domain of *r*:

(i) Form III: When all the three wave functions are nonzero in a domain, they must have the unique form as
  5 $${u}_{l}^{2}/{r}^{2}={X}_{l}-{Y}_{l}{r}^{2}$$
  where
  6 $${X}_{l}={{\mathfrak{D}}}_{{X}_{l}}/{\mathfrak{D}}$$
  ${{\mathfrak{D}}}_{{X}_{l}}$ is a determinant obtained by changing the *l* column of ${\mathfrak{D}}$ from (*α*_1*l*_, *α*_2*l*_, *α*_3*l*_) to (*ε*_1_, *ε*_2_, *ε*_3_).
  7 $${Y}_{l}={{\mathfrak{D}}}_{{Y}_{l}}/{\mathfrak{D}}$$
  ${{\mathfrak{D}}}_{{Y}_{l}}$ is also a determinant obtained by changing the *l* column of ${\mathfrak{D}}$ to (1/2,1/2,1/2). Once all the parameters are given, the three *Y*_*l*_ are known because they depend only on *α*_*ll*′_. However, the three *X*_*l*_ have not yet been known because they depend also on (*ε*_1_, *ε*_2_, *ε*_3_). When *Y*_*l*_ is positive (negative), *u*_*l*_/*r* goes down (up) with *r*. This point is notable because the main feature of the formal solution depends on the signs of {*Y*_*l*_}.
  The set {*X*_*l*_} and the set {*ε*_*l*_} are related as
  8 $${\varepsilon }_{l}={{\rm{\Sigma }}}_{l^{\prime} }{\alpha }_{ll^{\prime} }{X}_{l^{\prime} }$$
  9 $${X}_{l}={{\rm{\Sigma }}}_{l^{\prime} }{\bar{\alpha }}_{ll^{\prime} }{\varepsilon }_{l^{\prime} }$$
  where ${\bar{\alpha }}_{ll^{\prime} }={{\mathfrak{d}}}_{l^{\prime} l}/{\mathfrak{D}}$, and ${{\mathfrak{d}}}_{l^{\prime} l}$ is the algebraic cominor of *α*_*l*′*l*_.
(ii) Form II: When one and only one of the wave functions is zero inside a domain (say, *u*_*n*_/*r* = 0), the other two must have the unique form as
  10 $$\begin{array}{rcl}{u}_{l}^{2}/{r}^{2} & = & {X}_{l}^{(n)}-{Y}_{l}^{(n)}{r}^{2}\\ {u}_{m}^{2}/{r}^{2} & = & {X}_{m}^{(n)}-{Y}_{m}^{(n)}{r}^{2}\end{array}$$
  where *l*, *m*, and *n* are in a cyclic permutation of 1-2-3 (the same in the follows),
  11 $$\begin{array}{rcl}{X}_{l}^{(n)} & = & ({\alpha }_{mm}{\varepsilon }_{l}-{\alpha }_{lm}{\varepsilon }_{m})/{{\mathfrak{d}}}_{nn}\\ \,{Y}_{l}^{(n)} & = & \frac{1}{2}({\alpha }_{mm}-{\alpha }_{lm})/{{\mathfrak{d}}}_{nn}\\ {X}_{m}^{(n)} & = & ({\alpha }_{ll}{\varepsilon }_{m}-{\alpha }_{ml}{\varepsilon }_{l})/{{\mathfrak{d}}}_{nn}\\ \,{Y}_{m}^{(n)} & = & \frac{1}{2}({\alpha }_{ll}-{\alpha }_{ml})/{{\mathfrak{d}}}_{nn}\end{array}$$
  Once the parameters are given, the six ${Y}_{n^{\prime} }^{(n)}$ (*n*′ ≠ *n*) are known, while the six ${X}_{n^{\prime} }^{(n)}$ have not yet. When ${Y}_{n^{\prime} }^{(n)}$ is positive (negative), ${u}_{n^{\prime} }/r$ goes down (up) with *r*. When the Form II has *u*_*n*_/*r* = 0, a more precise notation Form II_*n*_ is adopted for the detail.
(iii) Form I: When one and only one of the wave functions is nonzero in a domain (say, *u*_*l*_/*r* ≠ 0), it must have the unique form as
  12 $${u}_{l}^{2}/{r}^{2}=\frac{1}{{\alpha }_{ll}}({\varepsilon }_{l}-{r}^{2}\mathrm{/2)}$$
  Obviously, *u*_*l*_/*r* in this form must descend with *r*. For the case *u*_*l*_/*r* ≠ 0, the more precise notation Form I_*l*_ is adopted.
(iv) Form 0: In this form all the three wave functions are zero.

If a wave function (say, *u*_*l*_/*r*) is nonzero in a domain but becomes zero when *r* = *r*_*o*_, then a downward form-transition (say, from Form III to II) will occur at *r*_*o*_. Whereas if *u*_*l*_/*r* is zero in a domain but becomes nonzero when *r* = *r*_*o*_, then a upward form-transition (say, from Form II to III) will occur at *r*_*o*_. *r*_*o*_ is named a form-transition-point, and it appears as the boundary separating the two connected domains. In this way the formal solutions serve as the building blocks, and they will link up continuously to form an entire solution. They must be continuous at the form-transition-points because the wave functions satisfy exactly the same set of nonlinear equations at those points. However, their derivatives are in general not continuous at the boundaries.

## An approach for obtaining analytical solutions of the CGP

In this section we consider the case that all the parameters are given and the values of the three {*ε*_*l*_} have been presumed. In this case all the formal solutions are known. We will propose an approach to link up the formal solutions to form a chain as a candidate of an entire solution. To this aim we first introduce a number of features related to the linking.

(i) For Form I to III, at least one of the wave function is descending with *r*.
  The proof of this feature is referred to ref. 36, where it is proved that at least one of *Y*_*l*_ (or ${Y}_{l}^{(n)}$ for a given *n*) is positive.
  This feature implies that, when *r* increases, the occurrence of a downward form-transition is inevitable, unless a upward form-transition takes place prior to the downward transition. In any cases a formal solution must transform to another form somewhere (except Form 0).
(ii) For a formal solution existing in a domain, the right boundary of the domain and the successor (the successive formal solution) in the next domain have been prescribed when the three {*ε*_*l*_} have been presumed.
  To prove this feature, as an example, we assume that *u*_1_/*r* and *u*_3_/*r* are nonzero in a domain while *u*_2_/*r* is zero. This assumption implies that we have assumed ${X}_{1}^{\mathrm{(2)}}-{Y}_{1}^{\mathrm{(2)}}{r}^{2}\ge 0$ and ${X}_{3}^{\mathrm{(2)}}-{Y}_{3}^{\mathrm{(2)}}{r}^{2}\ge 0$ when *r* is given inside the domain (refer to eq. (10)). We define ${r}_{1}^{2}={X}_{1}^{\mathrm{(2)}}/{Y}_{1}^{\mathrm{(2)}}$ or ∞ (if ${Y}_{1}^{\mathrm{(2)}} > 0$ or ≤ 0). Similarly, we define ${r}_{3}^{2}={X}_{3}^{\mathrm{(2)}}/{Y}_{3}^{\mathrm{(2)}}$ or ∞ (if ${Y}_{3}^{\mathrm{(2)}} > 0$ or ≤ 0), and ${r}_{2}^{2}={X}_{2}/{Y}_{2}$ or ∞ (if both *X*_2_ and *Y*_2_ are negative or otherwise). Then, the smallest one among *r*_1_, *r*_2_, and *r*_3_ is just the right boundary of the domain. Say, if *r*_1_ is the smallest, then *u*_1_/*r* → 0 when *r* → *r*_1_, and the successor will have the Form I_3_. If *r*_2_ is the smallest, then *u*_2_/*r* will emerge at *r*_2_, and the successor will have the Form III, and so on. Since *r*_1_, *r*_2_, and *r*_3_ are prescribed, the right boundary and the successor are prescribed
(iii) Once the formal solution in the first domain (starting from *r* = 0) is prescribed, the formal solutions will link up one-by-one to form a chain in a unique way. There are seven types of formal solutions (say, in Form II_2_, or in Form I_3_, and so on). Each type can appear in a chain at most once.
  Obviously, since the successor in each step of linking is uniquely prescribed, the whole chain is prescribed. Since the right boundary of a type is prescribed, the type can not appear twice.
(iv) Once a formal solution in a chain is in Form 0, the chain will end.

This is because no wave functions can emerge from an empty domain. Otherwise, if *u*_1_/*r* emerges alone, it must have the form as eq. (12). This form prohibits the uprising of *u*_1_/*r*. If *u*_1_/*r* and *u*_2_/*r* emerge at the same place, ${Y}_{1}^{\mathrm{(3)}}$ and ${Y}_{2}^{\mathrm{(3)}}$ must both be negative. This violates the feature (i). If all the {*u*_*l*_/*r*} emerge at the same place, all the three {*Y*_*l*_} must be negative. This violates also the feature (i).

Based on the above features, we propose an approach as follows: First, we design a chain for a type of entire solutions denoted as, for an example, II_2_-III-II_1_-I_3_ (it implies that the first domain has a Form II_2_, the next domain has a Form III, the third domain has a Form II_1_, while the last domain has a Form I_3_). The prescription on the linking appears as a number of requirements (inequalities) imposing on the W-strengths and the presumed {*ε*_*l*_}. When all the $\{{\alpha }_{ll^{\prime} }\}$ and the {*ε*_*l*_} are given inside a specific scope, all the requirements can be met and the designed chain as a candidate can be achieved. At this stage the normalization has not yet been considered. When the three equations $\int \,{u}_{l}^{2}dr=1$ are further introduced, not only the scope but the values of the set {*ε*_*l*_} can be fixed. Then, the candidate will be a realistic entire solution of the CGP. In general, the three equations can uniquely determine the three unknowns {*ε*_*l*_}, unless the design itself is not reasonable. Thus, when the parameters are given in a reasonable scope, we can uniquely find out a realistic entire solution, which is a chain of formal solutions with a specific linking. A detailed practice of this approach for miscible states is given in ref. 36.

## State-transition and the singularity of the matrix

Based on the above approach, numerical calculations for two types of examples are performed. Related wave functions are plotted.

1. State-transition occurring at the singular point of the matrix-of-equations

**Figure 1:**
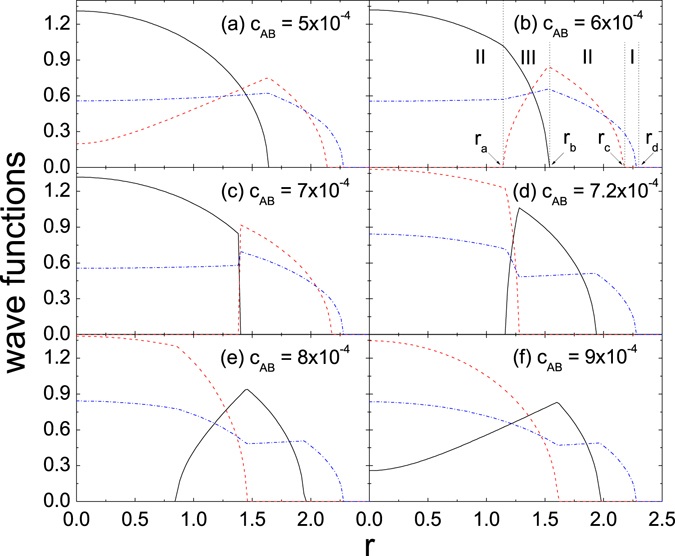
Wave functions *u*_1_/*r* (in solid line), *u*_2_/*r* (in dash line), and *u*_3_/*r* (in dash-dot line) against *r*. *ħω* and $\lambda \equiv \sqrt{\hslash /({m}_{o}\omega )}$ are used as units for energy and length. *c*_*AB*_ is given at six values marked in the panels. Other parameters are fixed and are chosen quite arbitrary but having *p*_*BC*_ and *p*_*CA*_ both being negative. They are *N*_*A*_ = 30000, *N*_*B*_ = 11000, *N*_*C*_ = 29000, *c*_*A*_ = 4 × 10^−4^ (in *ħωλ* ^3^, the same for other strengths), *c*_*B*_ = 1.4 × 10^−3^, *c*_*C*_ = 1.2 × 10^−3^, *c*_*BC*_ = 3.8 × 10^−4^, *c*_*CA*_ = 4.2 × 10^−4^, *γ*_*A*_ = *γ*_*B*_ = *γ*_*C*_ = 1. In 1b, the Forms of the solutions are marked in the associated domains separated by the vertical dotted lines.

Figure 1(b–e) is for II_2_-III-II_1_-I_3_, while (a) and (f) are for III-II_1_-I_3_. From (a) to (f) *c*_*AB*_ is increasing while the other parameters remain unchanged. Thus these patterns demonstrate the effect of *c*_*AB*_.

For 1b as an example, the second domain is in Form III. For this form both {*X*_*l*_} and {*Y*_*l*_} are proportional to $\mathrm{1/}{\mathfrak{D}}$. Therefore, when ${\mathfrak{D}}\to 0$, the wave functions will become extremely steep and the second domain will become extremely narrow as shown in (c). It turns out that, when ${c}_{AB}=7.02\times {10}^{-4}\hslash \omega {\lambda }^{3}\equiv {c}_{AB}^{crit\mathrm{(3)}}$, the matrix-of-equations becomes singular and accordingly ${\mathfrak{D}}=0$. When *c*_*AB*_ is close to and crosses over this critical value (from (c) to (d)), {*Y*_*l*_} will suddenly change their signs. It implies a down-falling wave function suddenly becomes up-rising, and accordingly the whole pattern is changed greatly. This is definitely associated with a state-transition in which all the A-atoms suddenly jump from a core to a shell, while all the B-atoms jump in a reverse way as clearly shown in (c) and (d). Accompanying the great change, a remarkable increase in the total energy is expected (this expectation is confirmed below).

From the equality ${\mathfrak{D}}=0$, it is straight forward to obtain

13 $${c}_{AB}^{crit\mathrm{(3)}}=\frac{1}{{c}_{C}}({c}_{BC}{c}_{CA}\pm \sqrt{{p}_{BC}{p}_{CA}})$$

where

14 $${p}_{BC}\equiv {c}_{BC}^{2}-{c}_{B}{c}_{C}$$

Similarly, *p*_*CA*_ and *p*_*AB*_ can be defined by permuting the indexes.

Note that:

(i) When *p*_*BC*_ *p*_*CA*_ < 0, ${c}_{AB}^{crit\mathrm{(3)}}$ does not exist (i.e., the matrix will not become singular). Therefore, even a Form III is contained in a chain, the variation of *c*_*AB*_ does not assure the occurrence of the state-transition. Only if the other five strengths are so chosen that *p*_*BC*_ *p*_*CA*_ ≥ 0, the critical point could exist and the transition could occur.
(ii) ${c}_{AB}^{crit\mathrm{(3)}}$ deviates remarkably from the well known critical value ${c}_{AB}^{crit\mathrm{(2)}}=\sqrt{{c}_{A}{c}_{B}}$ for 2-BEC. Thus, the state-transition is remarkably affected by the influence of the third kind of atoms. However, if both *c*_*BC*_ → 0 and *c*_*CA*_ → 0 (i.e., the influence is removed), one can prove from eq. (13) that ${c}_{AB}^{crit\mathrm{(3)}}\to {c}_{AB}^{crit\mathrm{(2)}}$.
(iii) ${c}_{AB}^{crit\mathrm{(3)}}$ depends on the other five strengths but not on the particle numbers, trap frequencies, and masses. This feature is the same as what has found in 2-BEC. Thus, in an experiment, the variation of the parameters other than the strengths will not change the critical values.
(iv) The state-transitions caused by the variation of other strengths can be similarly deduced. For an example, for the intra-species interaction of the A-atoms, when *c*_*A*_ increases and arrives at a critical value

15 $${c}_{A}^{crit\mathrm{(3)}}=\frac{1}{{P}_{BC}}({c}_{CA}({c}_{AB}{c}_{BC}-{c}_{B}{c}_{CA})-{c}_{AB}({c}_{AB}{c}_{C}-{c}_{BC}{c}_{CA}))$$

the matrix will become singular and the transition will occur.

In summary, for an entire solution contains a Form III, when the variation of the strengths leads to a cross-over of the singular point of the matrix, a state-transition will occur. Since the singularity of the matrix is inherent in the CGP but not a product of the TFA, thus the occurrence of the state-transitions at the critical values holds beyond the TFA. In fact, in the earliest study of the 2-BEC, the instability in the neighborhood of the critical value ${c}_{AB}^{crit\mathrm{(2)}}=\sqrt{{c}_{A}{c}_{B}}$ (the singular point of the two-rank matrix) has been pointed out^8^.

(2) State-transition occurring at the singular point of a sub-matrix-of-equations

**Figure 2:**
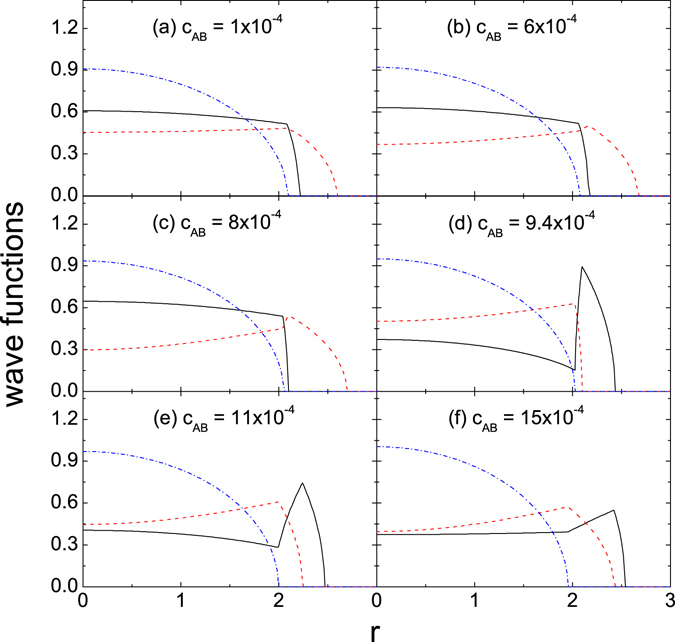
The same as in Fig. 1 but the parameters are so given that *p*_*BC*_ and *p*_*CA*_ are in opposite signs. The details of parameters are *N*_*A*_ = *N*_*B*_ = *N*_*C*_ = 30000, *c*_*A*_ = 4 × 10^−4^ (in *ħωλ* ^3^, the same in the follows), *c*_*B*_ = 2 × 10^−3^, *c*_*C*_ = 1 × 10^−3^, *c*_*BC*_ = 11.5 × 10^−4^, *c*_*CA*_ = 10.5 × 10^−4^, *γ*_*A*_ = *γ*_*B*_ = *γ*_*C*_ = 1.

When an entire solution contains a Form II, another type of state-transition might occur. In Fig. 2, the entire solution is III-II_3_-I_2_ in (a) to (c), and is III-II_3_-I_1_ in (d) to (f), where the second domain has the Form II_3_ (namely, only the A- and B-atoms are contained in this domain). For the Form II_3_ the critical value of *c*_*AB*_ is ${c}_{AB}^{crit\mathrm{(2)}}=\sqrt{{c}_{A}{c}_{B}}=8.944\times {10}^{-4}\hslash \omega {\lambda }^{3}$, which is the singular point of the sub-matrix of the equations for *u*_1_/*r* and *u*_2_/*r* only. When *c*_*AB*_ is close to this value ((c) and (d)) the second domain becomes very narrow, and the two wave functions become very steep. During the cross-over, ${Y}_{1}^{\mathrm{(3)}}$ and ${Y}_{2}^{\mathrm{(3)}}$ change their signs and a transition occurs as shown in (c) and (d). Nonetheless, different from the one found in Fig. 1, only a part of the A- and B-atoms are actively taking part in this transition, namely, a part of A-atoms rush out from the core and form a shell, while a part of outward B-atoms rush from the shell into the core. Thus, the corresponding change in spatial configuration is relatively milder. The change appears essentially in the second and the third domains where the C-atoms are absent. Accordingly, the critical value is not at all affected by the C-atoms and is identical to the value of 2-BEC. Incidentally, although the Form III is contained in Fig. 2, ${c}_{AB}^{crit\mathrm{(3)}}$ does not exist in this case due to *p*_*BC*_ *p*_*CA*_ < 0.

## Total energy of symmetric states and the great jump

When the total energy of the lowest symmetric state *E*_*tot*_ is higher than the total energy of the lowest asymmetric state ${E}_{tot}^{asym}$, the g.s. will be asymmetric. Thus, ${E}_{tot} > {E}_{tot}^{asym}$ is the discriminant to judge whether the g.s. is asymmetric.

**Figure 3:**
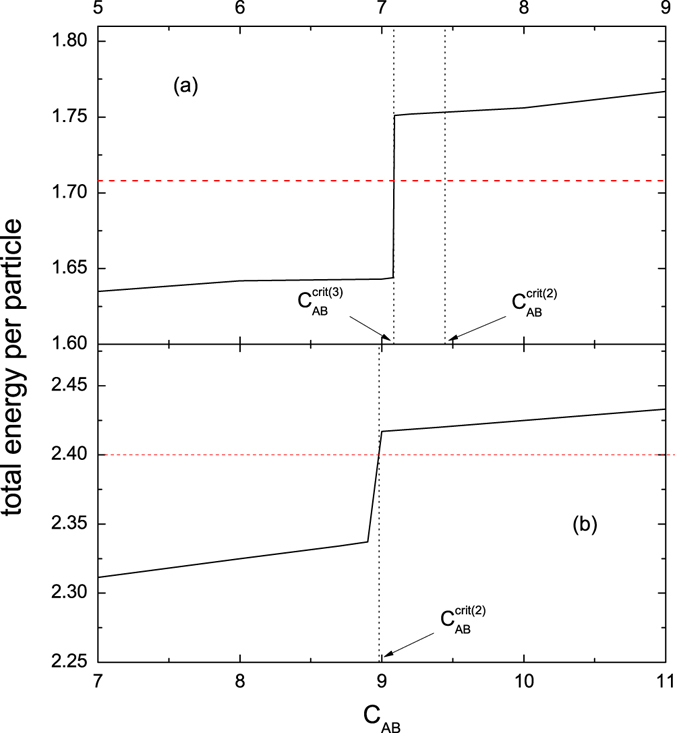
*E*_*tot*_/*N* (in solid line for the lowest symmetric state) and ${E}_{tot}^{asym}/N$ (in horizontal dash line for the lowest asymmetric state) versus *c*_*AB*_. Other parameters in (**a**,**b**) are the same as in Figs 1 and 2, respectively. The unit *ħω* is used for energy, and 10^−4^ *ħωλ* ^3^ for *c*_*AB*_. Note that the ranges of *c*_*AB*_ in (**a**,**b**) are different.

When the wave functions are known we can obtain the total energy as (the kinetic energy has been omitted)

16 $${E}_{tot}=\sum _{i}\,({P}_{i}+{E}_{i})+{{\rm{\Sigma }}}_{i < i^{\prime} }{E}_{ii^{\prime} }$$

where *i* = 1, 2 and 3. They are associated with *A*–, *B*–, and *C*– atoms, respectively. ${P}_{1}=\frac{{N}_{A}{\gamma }_{A}}{2}\int \,{u}_{1}^{2}{r}^{2}dr$, ${E}_{1}=\frac{{N}_{A}^{2}{c}_{A}}{8\pi }\int \,{({u}_{1}/r)}^{4}{r}^{2}dr$, ${E}_{12}=\frac{{N}_{A}{N}_{B}{c}_{AB}}{4\pi }\int \,{({u}_{1}/r)}^{2}{({u}_{2}/r)}^{2}{r}^{2}dr$, and so on. Let *N* = *N*_*A*_ + *N*_*B*_ + *N*_*C*_. Examples of *E*_*tot*_/*N* versus *c*_*AB*_ are plotted via the solid lines shown in Fig. 3. The other parameters in (a) and (b) are the same as in Figs 1 and 2, respectively. A distinguished feature is the appearance of the great jump at ${c}_{AB}^{crit\mathrm{(3)}}$ (a) and ${c}_{AB}^{crit\mathrm{(2)}}$ (b). Note that, in Fig. 3(a), the crossing over ${c}_{AB}^{crit\mathrm{(2)}}$ does not cause an effect because the associated transition could occur only if the chain contains the building block II_3_, this building block is absent in Fig. 1. While in Fig. 3(b) ${c}_{AB}^{crit\mathrm{(3)}}$ does not exist because *p*_*BC*_ *p*_*CA*_ < 0 as mentioned.

Figure 3 confirms that the state-transition has caused a great change in *E*_*tot*_. For the transition shown in Fig. 1(c,d), when the B-atoms rush in, *E*_2_ will increase (because a more compact distribution leads to the increase of the factor $\int \,{({u}_{2}/r)}^{4}{r}^{2}dr$) while *P*_2_ will remarkably decrease. The decrease over takes the increase. We found that, for each B-atom, (*E*_2_ + *P*_2_)/*N*_*B*_ decreases from 1.80 to 1.41. On the other hand, for each A-atom, (*E*_1_ + *P*_1_)/*N*_*A*_ increases from 1.08 to 1.50. Since ${N}_{A}\gg {N}_{B}$ in this example, totally, *E*_*tot*_ increases remarkably. This examples demonstrates that, although the critical value for the transition depends only on the strengths, the magnitude of the energy gap depends also on other parameters. The magnitude can be very large or quite small (say, in the above examples, the magnitude can be tuned by varying *N*_*A*_ and/or *N*_*B*_). Since all the A- and B- atoms are involved in the transition, the excitation is collective in nature.

## Asymmetric states and their total energy

We know from the study of the 2-BEC^9, 11, 12^ that, when *V*_*AB*_ is sufficiently strong, the A- and B-atoms might give up the symmetry of the trap for lowering the g.s. energy. Therefore, we propose a model where only the distributions of the A- and B-atoms are asymmetric, while the C-atoms are symmetric. Let *O* denotes the center of the trap. Let a sphere with radius *R*_*AB*_ centered at *O* be divided into two parts by a plane perpendicular to the Z-axis. The plane intersects the Z-axis at *z* = *z*_0_ (−*R*_*AB*_ < *z*_0_ < *R*_*AB*_). Let the A-atoms be distributed in the lower part of the sphere, and the B-atoms in the upper part. Let the C-atoms be symmetrically distributed in another sphere with radius *R*_*C*_ and centered also at *O*. Then, we assume

17 $${u}_{1}/r={d}_{1}\sqrt{1-{(r/{R}_{AB})}^{2}}$$

if *r* ≤ *R*_*AB*_ and *z* ≤ *z*_0_. Otherwise, it is zero. Where ${d}_{1}={(\frac{1}{15}{R}_{AB}^{3}+\frac{1}{8}{R}_{AB}^{2}{z}_{0}-\frac{1}{12}{z}_{0}^{3}+\frac{1}{40}{R}_{AB}^{-2}{z}_{0}^{5})}^{-\mathrm{1/2}}$ is for the normalization.

18 $${u}_{2}/r={d}_{2}\sqrt{1-{(r/{R}_{AB})}^{2}}$$

if *r* ≤ *R*_*AB*_ and *z* > *z*_0_. Otherwise, it is zero. Where ${d}_{2}={(\frac{1}{15}{R}_{AB}^{3}-\frac{1}{8}{R}_{AB}^{2}{z}_{0}+\frac{1}{12}{z}_{0}^{3}-\frac{1}{40}{R}_{AB}^{-2}{z}_{0}^{5})}^{-\mathrm{1/2}}$ and

19 $${u}_{3}/r={d}_{3}\sqrt{1-{(r/{R}_{C})}^{2}}$$

if *r* ≤ *R*_*C*_. Otherwise, it is zero. Where ${d}_{3}={(\frac{2}{15}{R}_{C}^{3})}^{-\mathrm{1/2}}$ When the values of *R*_*AB*_, *R*_*C*_, and *z*_0_ are assumed, from eqs (16, 17, 18 and 19), the total energy for the asymmetric state (with the kinetic energies neglected), denoted as ${E}_{tot}^{asym}$, can be obtained. The parameters *R*_*AB*_, *R*_*C*_, and *z*_0_ are considered as variable. Eventually, they fixed at the values that lead to the minimum of ${E}_{tot}^{asym}$. The ${E}_{tot}^{asym}$ obtained via such a variational procedure is in general higher than its actual value. Thus, in any cases, if we found ${E}_{tot}^{asym} < {E}_{tot}$, the asymmetric state will inevitably replace the symmetric g.s.

The comparison of the two energies are shown in Fig. 3, where (a) and (b) are associated with Figs 1 and 2, respectively.

Figure 3a demonstrates clearly that ${E}_{tot}^{asym}$ is remarkably lower than *E*_*tot*_ when ${c}_{AB} > {c}_{AB}^{crit\mathrm{(3)}}$. Thus the jump provides a good opportunity for the lowest asymmetric state to replace the symmetric g.s. Whereas when ${c}_{AB} < {c}_{AB}^{crit\mathrm{(3)}}$, although ${E}_{tot}^{asym}$ is remarkably higher than *E*_*tot*_ as shown in the figure and *E*_*tot*_ will decrease further with the decrease of *c*_*AB*_, we can only say that the g.s. is very probable to be symmetric. This is a point to be further studied.

## Division of the parameter-space

If the whole parameter-space Σ, in which a point is associated with a set of parameters, can be divided into zones each supports a specific configuration, various phase-diagrams could be plotted. Thereby the essential features of the system and the effects of the parameters can be visualized. Due to having so many parameters, the phase-diagrams of a 3-BEC would be very complicated. At this moment we are not able to plot them. The following is a primary attempt along this line.

There are four well defined and important surfaces in Σ. They are expressed via the equations ${\mathfrak{D}}=0$ and ${{\mathfrak{d}}}_{ii}=0$ (*i* = 1 to 3). In other words, each surface is an aggregation of a kind of singular points. We have proved under the TFA that a crossing over these surfaces may cause a state-transition and accordingly an increase of *E*_*tot*_. When the TFA is removed, in a domain of *r* in which all the {*u*_*i*_/*r*} are nonzero, the exact CGP can be written as

20 $$(\begin{array}{c}{u}_{1}^{2}/{r}^{2}\\ {u}_{2}^{2}/{r}^{2}\\ {u}_{3}^{2}/{r}^{2}\end{array})=\frac{1}{{\mathfrak{D}}}(\begin{array}{c}{{\mathfrak{d}}}_{11},{{\mathfrak{d}}}_{21},{{\mathfrak{d}}}_{31}\\ {{\mathfrak{d}}}_{12},{{\mathfrak{d}}}_{22},{{\mathfrak{d}}}_{32}\\ {{\mathfrak{d}}}_{13},{{\mathfrak{d}}}_{23},{{\mathfrak{d}}}_{33}\end{array})(\begin{array}{c}{\varepsilon }_{1}-\frac{{r}^{2}}{2}+\frac{1}{2}{(\frac{{m}_{o}\omega }{{m}_{A}{\omega }_{A}})}^{2}\frac{{u}_{1}^{{}^{{\rm{^{\prime} }}{\rm{^{\prime} }}}}}{{u}_{1}}\\ {\varepsilon }_{2}-\frac{{r}^{2}}{2}+\frac{1}{2}{(\frac{{m}_{o}\omega }{{m}_{B}{\omega }_{B}})}^{2}\frac{{u}_{2}^{{}^{{\rm{^{\prime} }}{\rm{^{\prime} }}}}}{{u}_{2}}\\ {\varepsilon }_{3}-\frac{{r}^{2}}{2}+\frac{1}{2}{(\frac{{m}_{o}\omega }{{m}_{C}{\omega }_{C}})}^{2}\frac{{u}_{3}^{{}^{{\rm{^{\prime} }}{\rm{^{\prime} }}}}}{{u}_{3}}\end{array})$$

where ${u}_{i}^{^{\prime\prime} }$ is the second-order derivative of *u*_*i*_ against *r*. The appearance of the common factor $\mathrm{1/}{\mathfrak{D}}$ at the right side implies that the left-side (namely, the wave functions) is extremely sensitive against the parameters when they are given in the neighborhood of the surface ${\mathfrak{D}}=0$. This is an important feature of the CGP. When a point in Σ crosses over ${\mathfrak{D}}=0$, the factor $\mathrm{1/}{\mathfrak{D}}$ changes from $\mp$∞ to ±∞. Therefore, the entire solutions (if it contains a Form III) will undergo a dramatic change, and the occurrence of the state-transition (found before under the TFA, refer to Fig. 1) is inevitable Thus, this kind of transition is inherent in the CGP. For the kind of entire solutions containing a Form III, once the variation of the parameters leads to a crossing over the surface ${\mathfrak{D}}=0$, the transition (denoted as trans-III) happens definitely.

Similarly, in a domain of *r* in which *u*_*n*_/*r* = 0, the exact CGP can be written in a form in which both (*u*_*l*_/*r*)^2^ and (*u*_*m*_/*r*)^2^ are proportional to a common factor $\mathrm{1/}{{\mathfrak{d}}}_{nn}$. Thus, for the type of entire solutions containing a Form II_*n*_, the crossing over the surface ${{\mathfrak{d}}}_{nn}=0$ will also lead to a great change in *u*_*l*_/*r* and *u*_*m*_/*r*, and accordingly another kind of state-transition (denoted as trans-II*n*) occurs as shown in Fig. 2.

Let us define a subspace Σ_*III*_ as follows. When a set of parameters leads to an entire solution containing a Form III, then the associated point belongs to Σ_*III*_, otherwise belongs to its complement. Let the part of the surface ${\mathfrak{D}}=0$ located inside Σ_*III*_ be denoted as *σ*_*III*_. Then, *σ*_*III*_ appears as a boundary, the crossing over this boundary leads to the trans-III. Similarly, let ${{\rm{\Sigma }}}_{I{I}_{3}}$ denotes the subspace containing the points each leads to an entire solution containing a Form II_3_. Let the part of the surface ${{\mathfrak{d}}}_{nn}=0$ located inside the subspace ${{\rm{\Sigma }}}_{I{I}_{3}}$ be denoted as ${\sigma }_{I{I}_{3}}$. Then, the crossing over ${\sigma }_{I{I}_{3}}$ leads to the transition trans-II_3_. We can further define ${\sigma }_{I{I}_{1}}$ and ${\sigma }_{I{I}_{2}}$ in a similar way. These four surfaces (*σ*_*III*_ and the three ${\sigma }_{I{I}_{i}}$) together form the boundaries and provide a primitive division of Σ. At the two sides of each boundary, the entire solutions are greatly different.

Nonetheless, these boundaries are not the actual boundaries for the phase-diagrams of the g.s. The latter can be made certain only if exact calculations on both the lowest symmetric and asymmetric states have been performed. However, since the crossing over the above boundaries leads to an increase of *E*_*tot*_ and the increase may be large (as shown in Fig. 3). Thus the increase provides an excellent opportunity for the lowest asymmetric state to replace the lowest symmetric state and become the g.s. Therefore, we believe that the exact boundaries for the phase diagrams would partially overlap the boundaries from singularity.

## Final remarks

1. A general approach is proposed to solve the CGP for 3-BEC in an analytical way. TFA has been adopted. The essence of this approach is to find out the building blocks, i.e., the formal solutions, and the rules for their linking. The entire solutions of the CGP appear as a chain of them. This approach is applicable for obtaining solutions with their chains in various types, and can be generalized to K-BEC with K larger than three. For examples, in a domain where all the K {*u*_*l*_/*r*} are nonzero, the formal solution has exactly the same expressions as shown in eqs (5, 6 and 7) except that the related matrixes are K-rank.
2. The main result of this paper is the finding of the state-transitions caused by the singularity of the (sub)matrix-of-equation and the associated increase of *E*_*tot*_ during the transition. The singularity is not a by-product of the TFA, but an important feature inherent in the CGP. Note that the critical behavior of the multiband superconductors was found to be substantially affected by the interband coupling^31–33^. Similarly, the critical point for the state-transition found in this paper differs remarkably from the one of the 2-BEC (refer to Fig. 1) due to the inter-species coupling. Note that the 3-BEC contains three subsystems, each contains two species. Similar to the hidden criticality found also in multiband superconductivity^32^, the critical points of the three subsystems appear as the hidden critical points of the 3-BEC. Under specific conditions state-transitions will also occur at these hidden points (refer to Fig. 2).
3. A model for asymmetric states has been proposed. Via numerical calculations on some examples, it is demonstrated that the lowest asymmetric state replaces the symmetric states and become the g.s. when the strength of an inter-species interaction arrives at and exceeds its critical value.
4. The whole parameter-space is primitively divided into zones separated by four surfaces as boundaries, each is an aggregation of a kind of singular points. The spatial configurations at the two sides of a boundary are greatly different due to the state-transition occurring during the crossing over the boundaries. The transition is accompanied with an energy increase, the amount of increase might be very large. Thus the state-transition provides an excellent opportunity for the emergence of the asymmetric g.s. Therefore, it is expected that the exact boundaries designating the zones of asymmetric g.s. would overlap partially with the boundaries arising from the singularity. This remains to be confirmed.
